# The effect of temperature and pressure on the crystal structure of piperidine

**DOI:** 10.1186/s13065-015-0086-3

**Published:** 2015-04-12

**Authors:** Laura E Budd, Richard M Ibberson, William G Marshall, Simon Parsons

**Affiliations:** EaStCHEM School of Chemistry and Centre for Science at Extreme Conditions, The University of Edinburgh, King’s Buildings, West Mains Road, Edinburgh, EH9 3FJ UK; ISIS Pulsed Neutron and Muon Facility, STFC Rutherford Appleton Laboratory, Harwell Science and Innovation Campus, Harwell Oxford, Didcot, OX11 0QX UK; Present address: Neutron Sciences Directorate, Oak Ridge National Laboratory, PO Box 2008 MS6475, Oak Ridge, TN 37831-6457 USA

## Abstract

**Background:**

The response of molecular crystal structures to changes in externally applied conditions such as temperature and pressure are the result of a complex balance between strong intramolecular bonding, medium strength intermolecular interactions such as hydrogen bonds, and weaker intermolecular van der Waals contacts. At high pressure the additional thermodynamic requirement to fill space efficiently becomes increasingly important.

**Results:**

The crystal structure of piperidine-d_11_ has been determined at 2 K and at room temperature at pressures between 0.22 and 1.09 GPa. Unit cell dimensions have been determined between 2 and 255 K, and at pressures up to 2.77 GPa at room temperature. All measurements were made using neutron powder diffraction. The crystal structure features chains of molecules formed by NH…N H-bonds with van der Waals interactions between the chains. Although the H-bonds are the strongest intermolecular contacts, the majority of the sublimation enthalpy may be ascribed to weaker but more numerous van der Waals interactions.

**Conclusions:**

Analysis of the thermal expansion data in the light of phonon frequencies determined in periodic DFT calculations indicates that the expansion at very low temperature is governed by external lattice modes, but above 100 K the influence of intramolecular ring-flexing modes also becomes significant. The principal directions of thermal expansion are determined by the sensitivity of different van der Waals interactions to changes in distance. The principal values of the strain developed on application of pressure are similarly oriented to those determined in the variable-temperature study, but more isotropic because of the need to minimise volume by filling interstitial voids at elevated pressure.

**Graphical Abstract:**
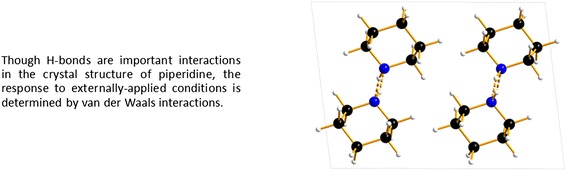
Though H-bonds are important interactions in the crystal structure of piperidine, the response to externally-applied conditions are determined by van der Waals interactions.

**Electronic supplementary material:**

The online version of this article (doi:10.1186/s13065-015-0086-3) contains supplementary material, which is available to authorized users.

## Background

**Scheme 1:**
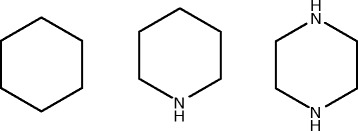
Cyclohexane, piperidine and piperazine.

Piperazine and piperidine are heterocyclic molecules related to cyclohexane by substitution of one or two CH_2_ groups by NH (Scheme 1). Cyclohexane, the ‘parent’ compound in the series, exhibits a rich diversity of phases, having been studied under varying degrees of temperature and pressure. Two phases are accessible by varying temperature alone. Phase I (space group $Fm\overline{3}m$), which occurs between the melting point (279.82 K) and 186.1 K, is a plastic phase in which the molecules undergo rapid molecular re-orientations about the lattice points. At 186.1 K the structure converts to an ordered phase II (space group *C*2/*c*) [1]. This transition, which is thermodynamically first order, has recently been investigated using total neutron scattering techniques to reveal formation of domains resembling phase-II as the phase transition temperature is approached from above [2]. Though the structure of phase-II is ordered, the distribution of molecular centroids retains the cubic-close packed topology of the plastic form.

In cyclohexane-II the molecules are distributed in layers which stack in the ABCABC… pattern characteristic of cubic close packing. In piperidine and piperazine the NH groups are capable of forming hydrogen bonds. In piperazine the arrangement of the molecular centroids is the same as in cyclohexane-II, but the molecules are rotated to enable H-bonds to form between the layers [3]. The structure of piperidine can also be considered to consist of layers, but the distribution of the molecules with respect to inversion centres in the space group makes the topology hexagonally close-packed. H-bonds form only between alternate layers, and the structure can be considered to be a hybrid of cyclohexane and piperazine. The rotations necessary for H-bond formation optimise interactions between the NH groups, but at the expense of breaking the van der Waals interactions formed within the layers of cyclohexane, and piperidine has a somewhat lower packing coefficient [4] (0.66) than either cyclohexane or piperazine (0.71).

The low packing coefficient suggests that piperidine may be susceptible to formation of different phases. This suggestion is supported by differential scanning calorimetry data presented by Parkin [3], which show that piperidine appears to form one phase on cooling from the liquid, which then transforms to another phase at 239 K. However, the higher temperature phase is not recovered on warming. In addition, during preliminary crystal growth experiments Parkin *et al.* obtained a weakly-diffracting form of piperidine with a cell volume approximately half of that of the phase they eventually characterised. The cell dimensions of this smaller-volume phase were *a* = 7.033(3), *b* = 5.224(3), *c* = 7.852(4) Å and β = 108.03(3)°. However, despite numerous attempts in which the cooling rate and sample size were varied this phase was never observed again.

The primary aim of the present investigation was therefore to determine if new forms of piperidine (in its perdeuterated form) could be identified under different conditions of temperature and pressure. The data collected also enable a detailed analysis of the thermodynamic properties of piperidine which are analysed with the aid of periodic DFT simulations and PIXEL packing energy calculations.

## Experimental

Piperidine-d_11_ was obtained from Aldrich and used as received.

### Variable temperature measurements

Variable temperature time-of-flight neutron powder diffraction data were recorded using the HRPD instrument at ISIS [5]. As piperidine is liquid under ambient conditions, piperidine-d_11_ was poured into a liquid nitrogen chilled stainless steel mortar [6] and cold-ground before being loaded into a rectangular aluminium sample can fitted with a heater. The sample was then placed in a cryostat held at 100 K. After confirming the sample was in the known phase, the temperature was reduced to 2 K and data collected at this temperature. Data were then collected in 5 K steps from 5 K to 250 K; there was no indication of a phase transition in this range. No evidence of a pre-melting transition was found on increasing the temperature further to 255 K.

In order to investigate the possibility of a second phase close to the melting point, the sample was removed from the cryostat and allowed to melt. It was then transferred to a cylindrical vanadium can containing glass wool (to promote formation of a randomly-oriented powder) before being placed in a cryostat held at 245 K. This should have been well above the transition temperature (239 K) reported in ref. [3], however upon crystallisation the known low temperature phase was obtained.

Quenching a similar sample of piperidine in liquid nitrogen also resulted in the sample crystallising into the same phase. No evidence for a second phase of piperidine was observed.

### High pressure measurements

Ambient temperature, high pressure neutron powder diffraction data were collected using the time-of-flight technique on the PEARL beamline high pressure facility at ISIS [7,8]. Piperidine-d_11_ was contained in a null-scattering Ti-Zr alloy capsule gasket [9] and loaded into a Paris-Edinburgh press [10-12]. The sample was loaded with powdered silica wool as previous work has shown it to aid formation of a well-oriented powder when crystallising liquids *in situ*; a small pellet of lead was included as a pressure marker. The pressure was calculated from the refined lead cell parameter using a Birch-Murnaghan equation of state [13,14] with *V*_0_ = 30.3128 Å^3^, *B* = 41.92 GPa, *B*’ = 5.72. These parameters were derived by Fortes [15,16] by refitting data obtained in three earlier studies [17-19].

On increasing the pressure to 0.22 GPa the sample crystallised in the monoclinic phase observed at low temperature. Diffraction data were collected at 0.22, 0.49, 0.80, 1.09 and 1.36 GPa for *ca* 700 μA h, with shorter (*ca* 100 μA h) collections at 2.06 and 2.77 GPa. Peak broadening was pronounced above 1.09 GPa, because of the lack of a hydrostatic medium, and therefore the sample was decompressed to approximately 0.23 GPa. Over a period of four hours the pressure decreased slightly resulting in the sample melting. Recompressing the sample to 0.30 GPa resulted in the sample recrystallising in the same phase.

### Structure refinement

**Figure 1:**
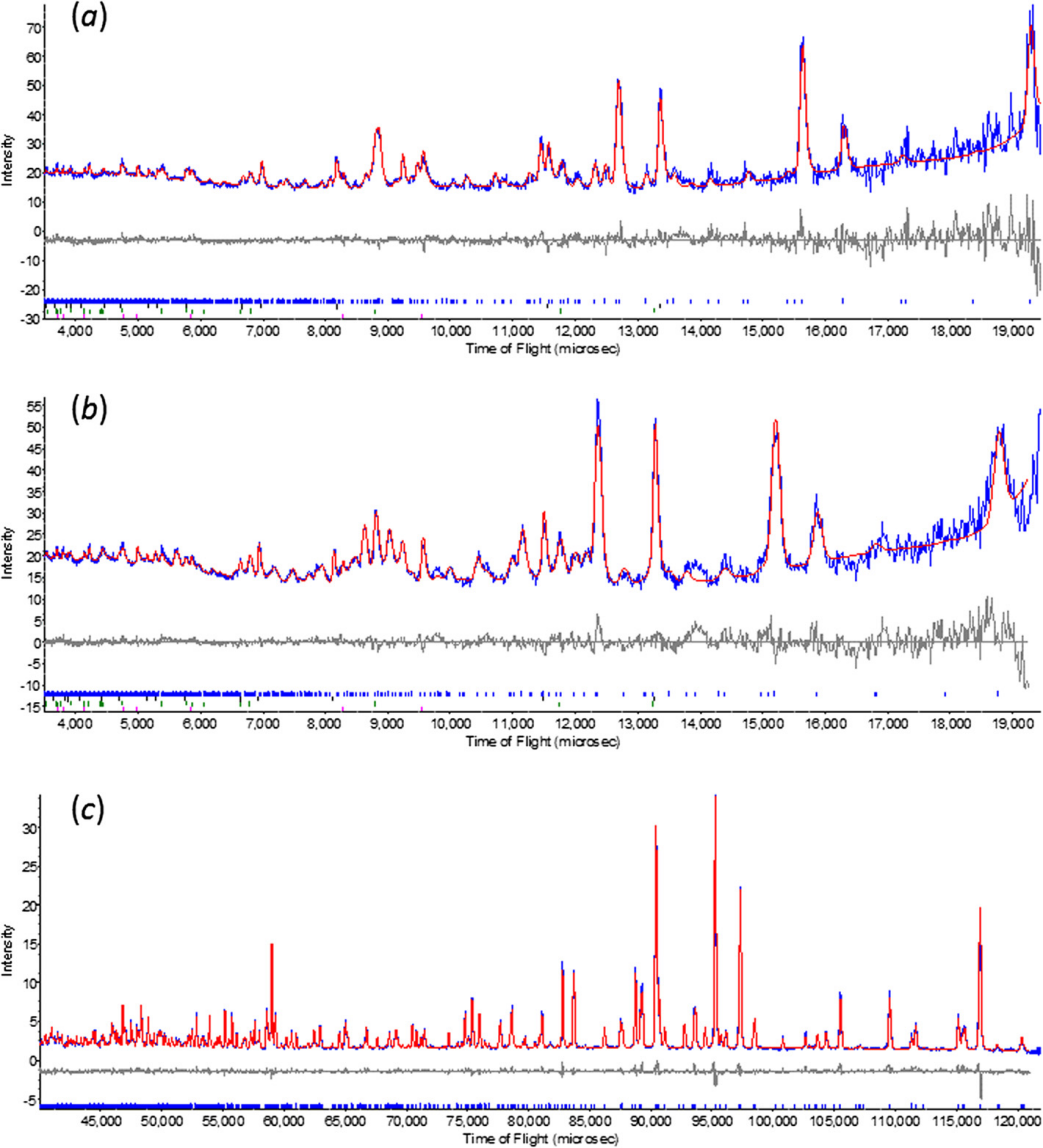
Rietveld refinement fits at (***a***) 0.22 GPa and room temperature, (***b***) 1.09 GPa and room temperature and (***c***) 2 K and ambient pressure. The *d*-spacing ranges are 0.73-4.16 Å for (***a***) and (***b***) and 0.83 – 2.75 Å for (***c***).

Rietveld refinements [20] were performed using TOPAS-Academic [21]. During refinement against the high-pressure data the piperidine molecules were treated as rigid groups using the *Z*-matrix formalism. Bond lengths and angles were taken from the previously determined structure (CSD refcode ITOBAU) [3] with deuterium distances set to standard neutron distances (N-H 1.009 Å, C-H 1.083 Å). Bond lengths, angles and torsion angles were not refined. All non-hydrogen atoms were refined with a common isotropic displacement parameter, as were all deuterium atoms. A fourth order spherical harmonic preferred orientation correction [22] was included as the sample was crystallised *in situ*. Ni and WC phases were also included in the refinement. The presence of these is due to the anvil cores of the Paris-Edinburgh press. Rietveld refinement profiles at 0.22 and 1.09 GPa are shown in Figure 1a and b, with crystal and refinement data given in Table 1. Peak broadening was quite pronounced above 1.09 GPa and detailed structure analysis was not carried out above this pressure.

**Table 1:** **Crystal and refinement data**

Pressure (GPa)	0.22	0.49	0.80	1.09	Ambient
Temperature (K)	298	298	298	298	2
Chemical formula	C_5_D_11_N	C_5_D_11_N	C_5_D_11_N	C_5_D_11_N	C_5_D_11_N
M_r_	96.17	96.17	96.17	96.17	96.17
Cell setting, space group	Monoclinic, *P*2_1_/*c*	Monoclinic, *P*2_1_/*c*	Monoclinic, *P*2_1_/*c*	Monoclinic, *P*2_1_/*c*	Monoclinic, *P*2_1_/*c*
*a, b, c* (Å)	8.6994(17), 5.2552(9), 11.9045(16)	8.5969(12), 5.2010(7), 11.7936(12)	8.5150(14), 5.1577(8), 11.6988(14)	8.4452(15), 5.1204(9), 11.6181(16)	8.59695(4), 5.21506(2), 11.93271(4)
α, β, γ (°)	90, 96.468(17), 90	90, 96.507(14), 90	90, 96.532(15), 90	90, 96.558(17), 90	90, 96.8790(4), 90
*V* (Å^3^)	540.77(16)	523.93(12)	510.45(13)	499.11(14)	531.135(4)
*Z*	4	4	4	4	4
D_calc_ (g cm^−3^)	1.181	1.219	1.251	1.280	1.203
Diffractometer	PEARL, ISIS				HRPD, ISIS
Collection method	Time of flight	Time of flight	Time of flight	Time of flight	Time of flight
Fitted range of d (Å)	0.75 – 4.17	0.75 – 4.17	0.75 – 4.17	0.75 – 4.12	0.83 – 2.51
R_p_ (%)	5.329	4.484	4.370	4.363	4.626
R_wp_ (%)	4.226	3.485	3.265	3.396	5.589
S	1.294	1.432	1.344	1.395	1.544
Background	9 term Chebychev polynomial				6 term Chebychev polynomial
Profile function	Back-to-back exponential convoluted with Voigt function				
Number of parameters	42	42	42	42	74

During refinement of the data collected at 2 K the piperidine molecules were treated using the *Z*-matrix formalism in TOPAS-Academic. Bond lengths and angles were refined, though all C-D distances were constrained to be equal. Anisotropic displacement parameters were modelled using the TLS formalism [23,24]. In spite of cold-grinding it was necessary to include a fourth order spherical harmonic correction for preferred orientation. The Rietveld refinement profile is shown in Figure 1c with crystal and refinement data given in Table 1. Pawley fits [25] were carried out on the rapid-scan data to obtain the lattice parameters.

### DFT calculations

DFT calculations were performed on piperidine-d_11_ using the plane-wave pseudopotential method in the CASTEP code [26] as incorporated into Materials Studio version 7 [27]. The PBE exchange-correlation functional [28] was used with norm-conserving pseudopotentials and a basis set cut-off energy of 950 eV. Brillouin zone integrations were performed with a Monkhorst-Pack [29] **k**-point grid spacing of 0.07 Å^−1^, corresponding to a grid of 2 × 3 × 1 points. These parameters gave an energy convergence of < 0.1 meV/atom.

For analysis of the effect of pressure and temperature on intermolecular interactions geometry optimisations were carried out while holding the unit cell parameters fixed to those observed experimentally. The total energy convergence tolerance was 5×10^−6^ eV/atom, with a maximum force tolerance of 0.01 eV Å^−1^ and a maximum displacement of 5×10^−4^ Å. The SCF convergence criterion was 5×10^−7^ eV/atom, and the space group symmetry was retained during geometry optimisation.

For frequency calculations, the geometry optimisation was carried out in two stages. The coordinates and unit cell dimensions were optimised using the Tkatchenko-Scheffler correction for dispersion (DFT-D) [30,31] starting from the experimentally determined structure determined at 2 K. The convergence criteria at this stage were the same as those given above with the additional requirement of a maximum stress tolerance of 0.02 GPa. The optimised cell parameters and volume were: *a* = 8.537417 [expt at 2 K: 8.59695(4)] Å, *b* = 5.169532 [5.21506(2)] Å, *c* = 11.923783 [11.93271(4)] Å, β = 96.834764 [96.8790(4)]° and *V* = 522.509783 [531.14(1)] Å^3^, reproducing the cell dimensions to within 1% and the volume to 1.6%. Linear response [32] calculations are not yet possible in CASTEP with dispersion-corrected functionals, and so a second stage of optimisation was carried out with the pure PBE functional holding the cell dimensions fixed. At this stage the energy convergence tolerance was 5×10^−7^ eV/atom, with a maximum force tolerance of 0.002 eV Å^−1^ and a maximum displacement of 5×10^−5^ Å; the SCF convergence criterion was 5×10^−10^ eV/atom. The maximum force on any atom at convergence was 0.00012 eV Å^−1^. Phonon density-of-states and dispersion curves were calculated with Fourier interpolation [33]. No imaginary frequencies were observed at any point in the Brillouin zone. Generation of structures distorted along phonon modes was accomplished with MODE_FOLLOW [34].

### PIXEL calculations

Electron densities were calculated using Gaussian09 [35] at the MP2 level of theory with the 6-31G** basis set using molecular geometries derived from the crystal structures. The PIXEL method [36-40], as implemented in the program OPiX [41], was then used to calculate the intermolecular interaction energies.

### Other programs used

Structures were visualised with XP [42], MERCURY [43] and DIAMOND [44]. Strain tensor calculations were carried out using a locally written program [45], based on the discussion by Ohashi and Burnham [46,47] and employing the JACOBI subroutine *Numerical Recipes* [48]. Equation-of-state calculations were performed using EOSfit [49]. ORIGIN was used for fitting of the unit cell parameters to variable temperature unit cell data. Animated GIFs were created from images generated in MERCURY using the web-site http://www.gifmaker.me/#003.

## Results and discussion

The crystal structure of piperidine has been previously determined from X-ray data at 150 K. Variable temperature neutron powder diffraction revealed the structure exists between 2 K and 255 K. Attempts to access another phase by crystallisations via rapid cooling to 245 K and 77 K were not successful. Crystallisation via the application of pressure resulted in the same phase as obtained at low temperature. No phase transitions were evident upon compression to 2.77 GPa.

### The crystal structure of piperidine

**Figure 2:**
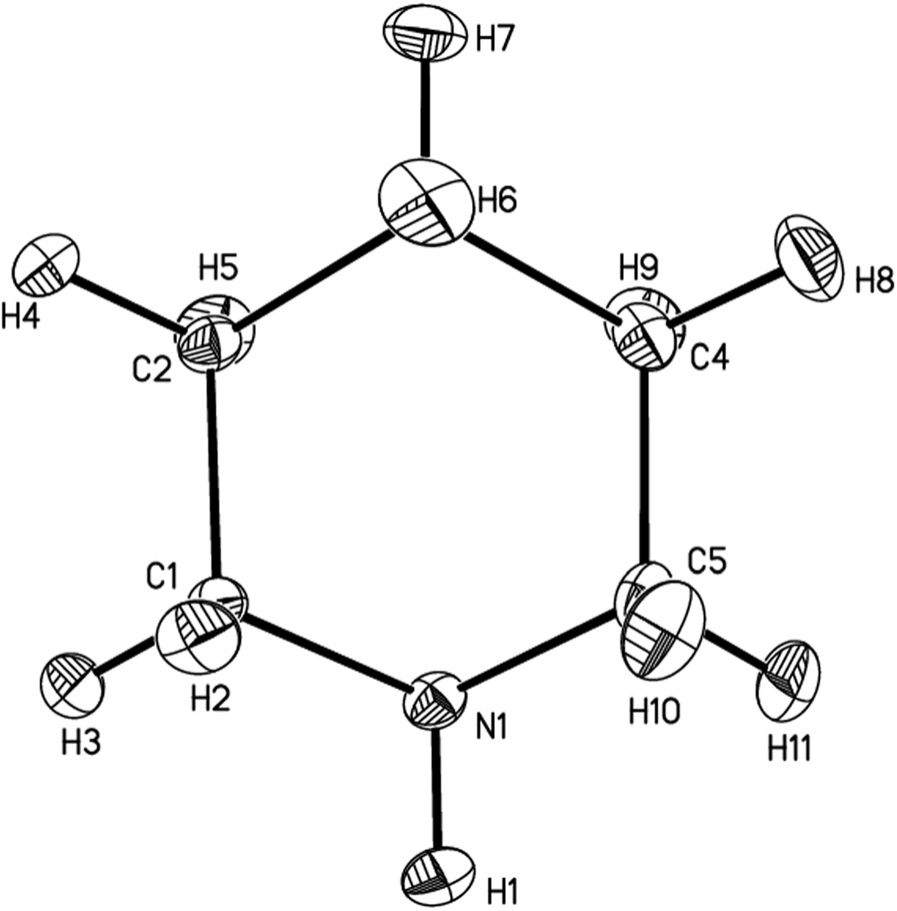
The molecular structure of piperidine at 2 K showing the displacement ellipsoids at the 50% probability level.

Piperidine crystallises in the monoclinic space group *P*2_1_/*c* with one molecule in the asymmetric unit (Figure 2) occupying a general position. Bond distances and angles adopt normal values as compared against similar structures in the Cambridge Database [50]. Ring-puckering analysis indicates that the molecule adopts an ideal chair conformation [51]. The hydrogen atom is attached to the nitrogen in the equatorial ring position.

**Figure 3:**
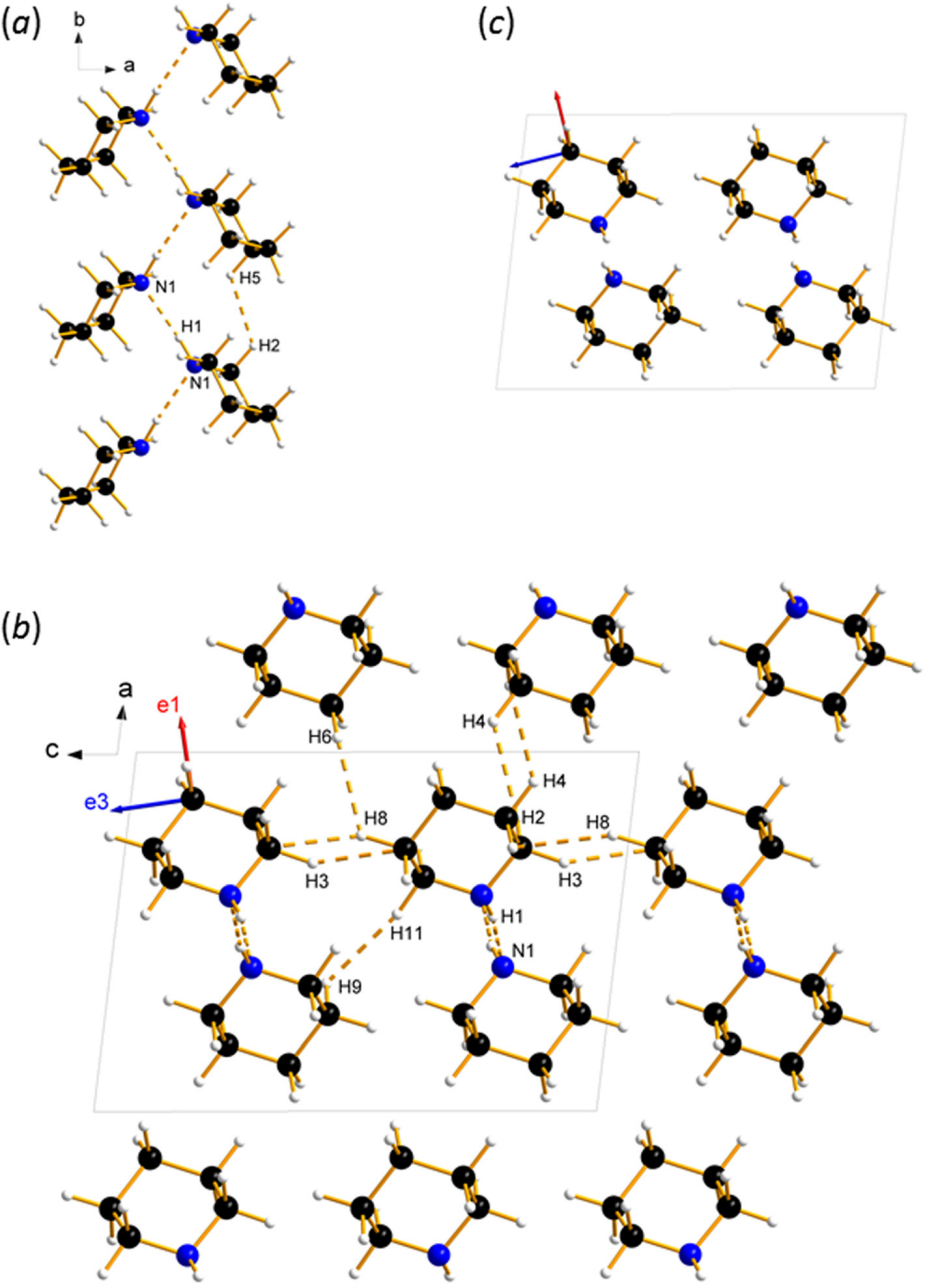
Intermolecular interactions in piperidine. (***a***) H-bonded chain at 2 K viewed along **c**. (***b***) View along **b** at 2 K. (***c***) View along **b** at 0.22 GPa. In (***a***) and (***b***) H…H contacts listed in Table 2 are also shown. In (***b***) and (***c***) the directions of the maximum and minimum eigenvalues of the strain tensor are shown in red and blue, respectively.

PIXEL calculations yield a value of 57 kJmol^−1^ for the sublimation enthalpy. The sublimation enthalpy at 262.12 K estimated from the experimentally-determined enthalpies of fusion [52] and vaporisation [53] and the heat capacities (*C*_p_) of the liquid [52] and gas [53] is also 57 kJ mol^−1^, in excellent agreement with the PIXEL value. For comparison, cyclohexane and piperazine have sublimation enthalpies of 37 and 72 kJmol^−1^, respectively [54]. The most significant intermolecular interaction is an N-H…N hydrogen bond [2.141(3) Å at 2 K] which links the molecules to form chains parallel to **b**, Figure 3a and Table 2. Successive molecules along the chains are related by the 2_1_ screw axis. The PIXEL calculations indicate that the H-bond energy is 23 kJ mol^−1^, accounting for about 40% of the calculated sublimation enthalpy. Ten more intermolecular contacts are formed with energies of between 8 and 4 kJ mol^−1^, with dispersion being the largest contributing term in each case (Table 2). Each of these contacts features a H…H contact distance of between 2.36 and 2.59 Å, *i.e.* at or slightly more than twice the sum of the of the van der Waals radius of H (2.4 Å). The contacts with energies of 8 kJmol^−1^ are formed to the next-nearest-neighbours within the H-bonded chains. The H-bonded chains are arranged so that the molecules of one chain fit into the grooves of the neighbouring chains (Figure 3b), and overall the twelve contacts are distributed with molecular centroids in the approximately close-packed arrangement identified in the original report of the structure. In Figure 3b the layers alluded to in Introduction run horizontally. The largest contact energy beyond the set of 12 contacts just described is 1.6 kJ mol^−1^.

**Table 2:** **Intermolecular contact distances in piperidine-d**_**11**_ **as determined by neutron powder diffraction, and molecule-molecule contact energies** ***E*** **estimated in PIXEL calculations**

**Contact**	**Symmetry operation**	**−** ***E***_**COUL**_ **/** **kJmol** ^**−1**^	**−** ***E***_**POL**_ **/** **kJmol** ^**−1**^	**−** ***E***_**DISP**_ **/** **kJmol** ^**−1**^	***E***_**REP**_ **/** **kJmol** ^**−1**^	**−** ***E***_**TOT**_ **/** **kJmol** ^**−1**^	**Distance/Å**	**Distance/Å**	**Distance/Å**
***T*** **or** ***P***							***T*** **= 2 K**	***P*** **= 0.22 GPa**	***P*** **= 1.09 GPa**
Contacts formed within H-bonded chains									
1. N1…H1-N1	[−x + 1,y − ½, −z + ½]	30.5	11.8	19.2	38.9	22.7	2.141(3)	2.18	2.09
2. N1-H1…N1	[−x + 1,y + ½, −z + ½]	30.5	11.8	19.2	39	22.6	2.141(3)	2.18	2.09
3. C1-H2…H5	[x,y + 1,z]	4.2	1.9	15.5	13.4	8.2	2.365(6)	2.41	2.27
4. C2-H5…H2	[x,y − 1,z]	4.2	1.9	15.5	13.4	8.2	2.365(6)	2.41	2.27
Contacts formed between H-bonded chains									
5. C2-H4…H2	[−x + 2,y − ½, −z + ½]	2.2	1.2	12.1	9.5	6.0	2.373(9)	2.42	2.31
6. C1-H2…H4	[−x + 2,y + ½, −z + ½]	2.2	1.2	12.1	9.5	6.0	2.373(9)	2.42	2.31
7. C4-H8…H6	[−x + 2, −y, −z + 1]	2.4	1.0	11.2	8.8	5.7	2.446(14)	2.49	2.38
8. C5-H11…H9	[−x + 1, −y, −z + 1]	3.0	1.5	10.5	9.7	5.4	2.382(13)	2.44	2.26
9. C5-H10…H3	[x, −y + ½,z + ½]	1.1	0.4	6.8	3.9	4.4	2.588(9)	3.04	2.51
10. C1-H3…H10	[x, −y + ½,z − ½]	1.1	0.4	6.8	3.9	4.4	2.588(9)	3.04	2.51
11. C4-H8…H5	[x, −y − ½,z + ½]	1.7	0.7	8.0	6.3	4.1	2.443(6)	2.51	2.33
12. C2-H5…H8	[x, −y − ½,z − ½]	1.7	0.7	8.0	6.3	4.1	2.443(6)	2.51	2.33

The subscripts COUL, POL, DISP, REP and TOT refer to Coulombic, polarisation, dispersion, repulsion and total energy terms. Distances from the high-pressure measurements are quoted without uncertainties because the piperidine molecules were treated as rigid bodies during refinement.

**Figure 4:**
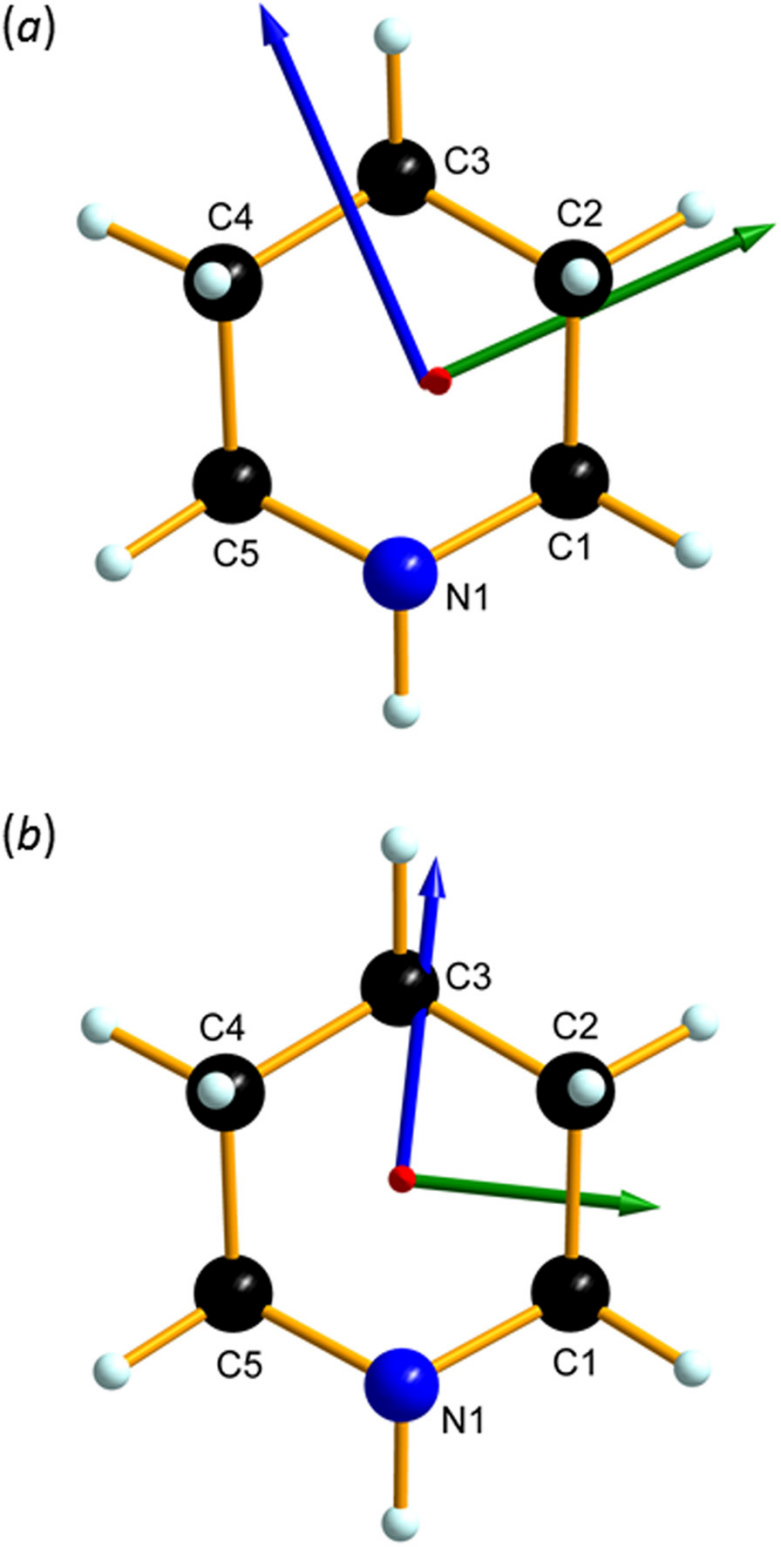
Largest eigenvalues of the rigid body thermal motion libration tensor *L*, determined (***a***) from experimental data at 2 K and (***b***) from a periodic DFT phonon calculation.

Anisotropic displacement parameters were modelled using TLS rigid-body constraints [23,24]. The two largest principal axes of the libration tensor, shown in Figure 4a are located in the mean plane of the molecule with magnitudes of approximately 19 degrees^2^, with the third, out-of-plane, axis having a magnitude of 4.2 degrees^2^. These data indicate that at 2 K the molecular motion consists mainly of in-plane rocking vibrations. This description of the molecular motion is broadly reproduced in the displacement parameters calculated from the periodic DFT phonon calculations (Figure 4b). The two in-plane *L* tensor eigenvalues have magnitudes of 12.0 and 15.0 degrees^2^, with the third axis having a value of 5.8 degrees^2^. The corresponding in-plane eigenvectors, though similar, are rotated by 29.6° relative to the experimental values. Considering the approximations made in the calculations (e.g. harmonicity), the nearly isotropic cross-section of the *L* tensor, the tendency for displacement parameters to ‘mop-up’ experimental errors such as absorption and the fact that the atomic motions are independent and not part of a rigid body in the DFT calculation, the overall the agreement between the experimental and theoretical results is quite satisfactory.

### The effect of temperature on the structure of piperidine

**Figure 5:**
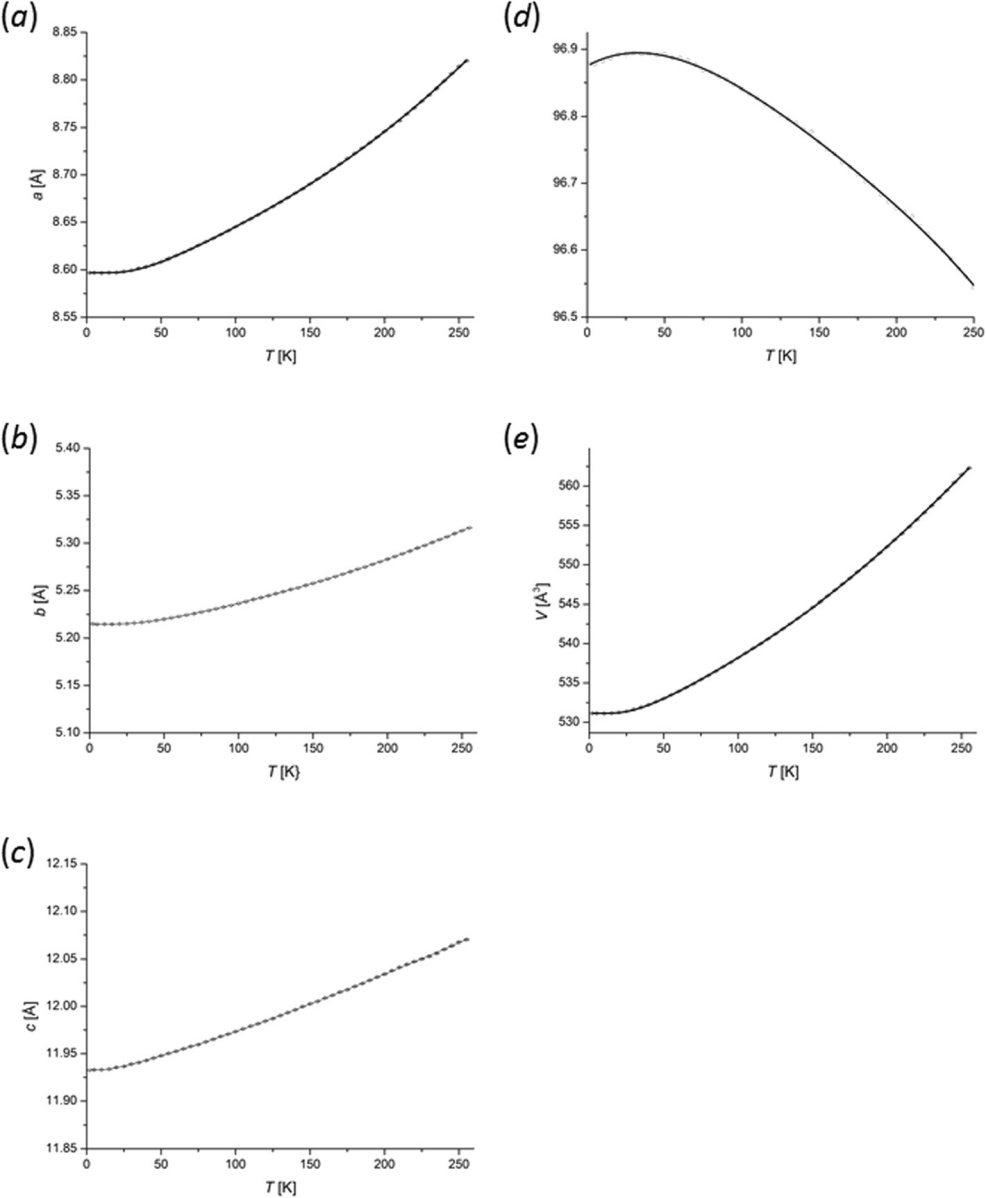
Variation of the unit cell dimensions with temperature. (***a***) the *a*-axis length, (***b***) the *b*-axis length, (***c***) the *c*-axis length, (***d***) the β angle and (***e***) the volume. The vertical axes in plots *a*-*c* span the same distance range.

The low temperature behaviour of piperidine was surveyed from 5 K to 255 K with data collected every 5 K. The extracted lattice parameters are given in the Additional file 1: Table S1 and displayed in Figure 5. The data exhibit a smooth expansion of the unit cell with no evidence of a phase transition.

The unit cell volume versus temperature data (Figure 5e) can be analysed following the methods described in refs [55-57]. This analysis starts with the definition of the Grüneisen ratio,γ [58,59],

1 $$ \gamma =\frac{\alpha \mathit{\mathsf{V}}\mathit{\mathsf{B}}}{{\mathit{\mathsf{C}}}_{\mathit{\mathsf{v}}}} $$

where α is the volume thermal expansion coefficient, *B* the isothermal bulk modulus, *V* is the unit cell volume and *C*_v_ the constant-volume heat capacity. Assuming both γ and *B* are independent of temperature, integration of [1] with respect to temperature gives

2 $$ \frac{\gamma }{\mathit{\mathsf{B}}}\left[\mathit{\mathsf{U}}\left(\mathit{\mathsf{T}}\right)-\mathit{\mathsf{U}}\left({\mathit{\mathsf{T}}}_{\mathsf{0}}\right)\right]=\mathit{\mathsf{V}}\left(\mathit{\mathsf{T}}\right)-\mathit{\mathsf{V}}\left({\mathit{\mathsf{T}}}_{\mathsf{0}}\right) $$

where *T*_0_ is a reference temperature and *U* refers to internal energy. Equation 2 demonstrates that the unit cell volume is a probe for changes in internal energy as a function of temperature.

The internal energy can be estimated using the Einstein model, Equ. 3, which assumes that all *N* atoms in the unit cell vibrate at the same frequency, ν, and the Einstein temperature θ = *hν/k*, where *h* is Planck’s constant.

3 $$ \mathit{\mathsf{U}}\left(\mathit{\mathsf{T}}\right)=\mathsf{3}\mathit{\mathsf{N}}\mathit{\mathsf{k}}\theta \left(\frac{\mathsf{1}}{\mathsf{2}}+\frac{\mathsf{1}}{ \exp \left(\theta /\mathit{\mathsf{T}}\right)-\mathsf{1}}\right) $$

Substitution of [3] into [2] gives

4 $$ \frac{\mathsf{3}\mathit{\mathsf{N}}\mathit{\mathsf{k}}\theta \gamma }{\mathit{\mathsf{B}}}\left(\frac{\mathsf{1}}{ \exp \left(\theta /\mathit{\mathsf{T}}\right)-\mathsf{1}}-\frac{\mathsf{1}}{ \exp \left(\theta /{\mathit{\mathsf{T}}}_{\mathsf{0}}\right)-\mathsf{1}}\right)=\mathit{\mathsf{V}}\left(\mathit{\mathsf{T}}\right)-\mathit{\mathsf{V}}\left({\mathit{\mathsf{T}}}_{\mathsf{0}}\right) $$

As *T*_0_ approaches zero the term 1/exp(θ/*T*_0_)-1 becomes small enough to be neglected, and

5 $$ \mathit{\mathsf{V}}\left(\mathit{\mathsf{T}}\right)=\mathit{\mathsf{V}}\left({\mathit{\mathsf{T}}}_{\mathsf{0}}\right)+\mathit{\mathsf{X}}\left(\frac{\mathsf{1}}{ \exp \left(\theta /\mathit{\mathsf{T}}\right)-\mathsf{1}}\right) $$

where

6 $$ \mathit{\mathsf{X}}=\frac{\mathsf{3}\mathit{\mathsf{N}}\mathit{\mathsf{k}}\theta \gamma }{\mathit{\mathsf{B}}} $$

Fitting of volume versus temperature data using equation (5) yields values of θ, *V*(*T*_0_) and *X* which can be interpreted in terms of vibrational, structural and heat capacity data.

Equation (5) was found to be inadequate for fitting the unit cell volume versus temperature data. Fitting to a ‘double-Einstein’ expression of the form

$$ \mathit{\mathsf{V}}\left(\mathit{\mathsf{T}}\right)=\mathit{\mathsf{V}}\left({\mathit{\mathsf{T}}}_{\mathsf{0}}\right)+{\mathit{\mathsf{X}}}_{\mathsf{1}}\left(\frac{\mathsf{1}}{ \exp \left({\theta}_{\mathsf{1}}/\mathit{\mathsf{T}}\right)-\mathsf{1}}\right)+{\mathit{\mathsf{X}}}_{\mathsf{2}}\left(\frac{\mathsf{1}}{ \exp \left({\theta}_{\mathsf{2}}/\mathit{\mathsf{T}}\right)-\mathsf{1}}\right) $$

was more successful (Figure 5e), yielding Einstein temperatures of 101.6(14) and 773(18) K (Table 3), which correspond to vibrational energies of *ca* 70 and 540 cm^−1^. The relatively large uncertainty on θ_2_ is a reflection of its value being well beyond the temperature range used for its determination. It was also possible to fit the unit cell lengths in the same way, and the results are depicted in Figure 5a-c, with parameters listed in Table 3. The angle β varies by little more than 0.3° (Figure 5d) over the temperature range studied, increasing very slightly between 2 and 50 K and decreasing thereafter. Einstein functions fail to model this trend, and for the purposes of interpolation the values were fitted to a quartic polynomial.

**Table 3:** **Einstein model fitting parameters**

	***X***_**0**_ **/Å or Å** ^**3**^	***X***_**1**_ **/Å or Å** ^**3**^	***X***_**2**_ **/Å or Å** ^**3**^	***Θ***_**1**_ **/K**	***Θ***_**2**_ **/K**
*V*	531.142(13)	12.4(3)	116(7)	101.6(14)	773(18)
*a*	8.59703(13)	0.108(3)	1.52(16)	118(2)	936(33)
*b*	5.21490(4)	0.0481(12)	0.40(3)	117.1(17)	791(22)
*c*	11.93275(9)	0.0246(16)	0.090(8)	48(2)	431(33)

The variation in the angle β was modelled with the equation β = 96.8752(10) + 0.126(7)*T*‘ − 0.231(12)*T*’^2^ + 0.084(7)*T*‘^3^ − 0.0130(15)*T‘* ^4^, where *T*’ = *T*/100.

**Figure 6:**
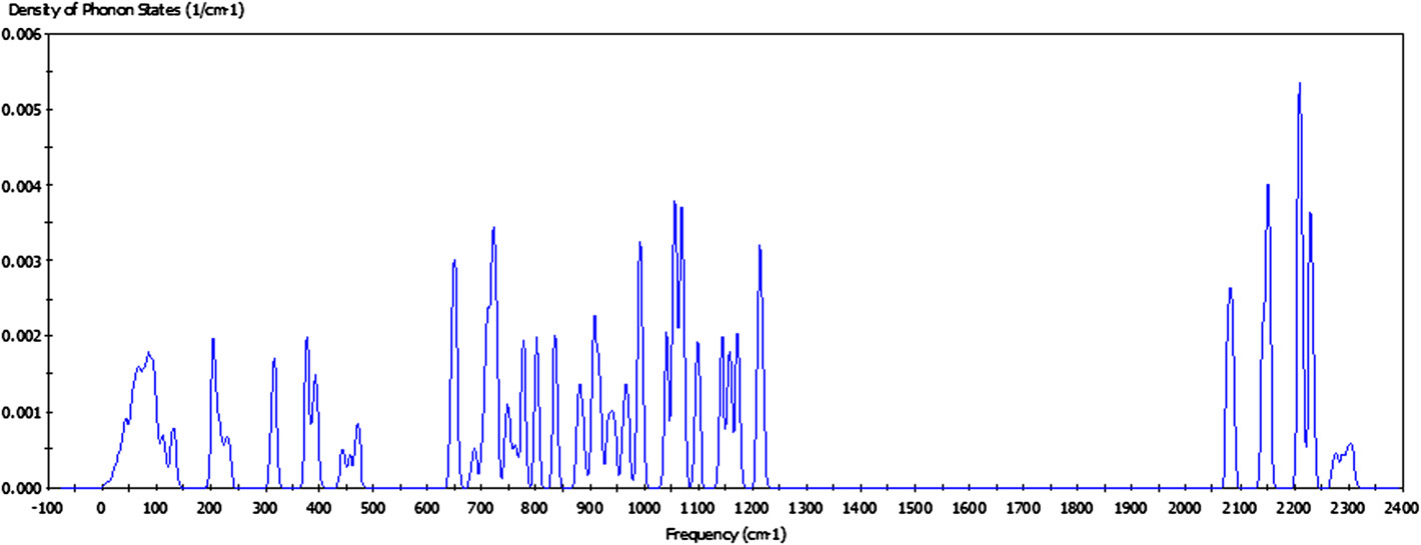
Phonon density of states of piperidine-d_11_ calculated by periodic DFT.

The Einstein temperatures extracted from the volume versus temperature data are in reasonable agreement with values determined from heat capacities (*C*_v_) simulated as a function of temperature using the results of the DFT phonon calculations (121 and 801 K). The phonon density of states plot shown in Figure 6 suggests that the single-frequency model was unsuccessful because the low-frequency phonon region divides into one block at about 100 cm^−1^ composed of intermolecular modes, and a second from 200–500 cm^−1^ corresponding to intramolecular torsional modes such as ring-flexing. Both sets of vibrations contribute to the internal energy between 2 and 255 K, and both therefore need to be taken into account in the data-fitting model.

**Figure 7:**
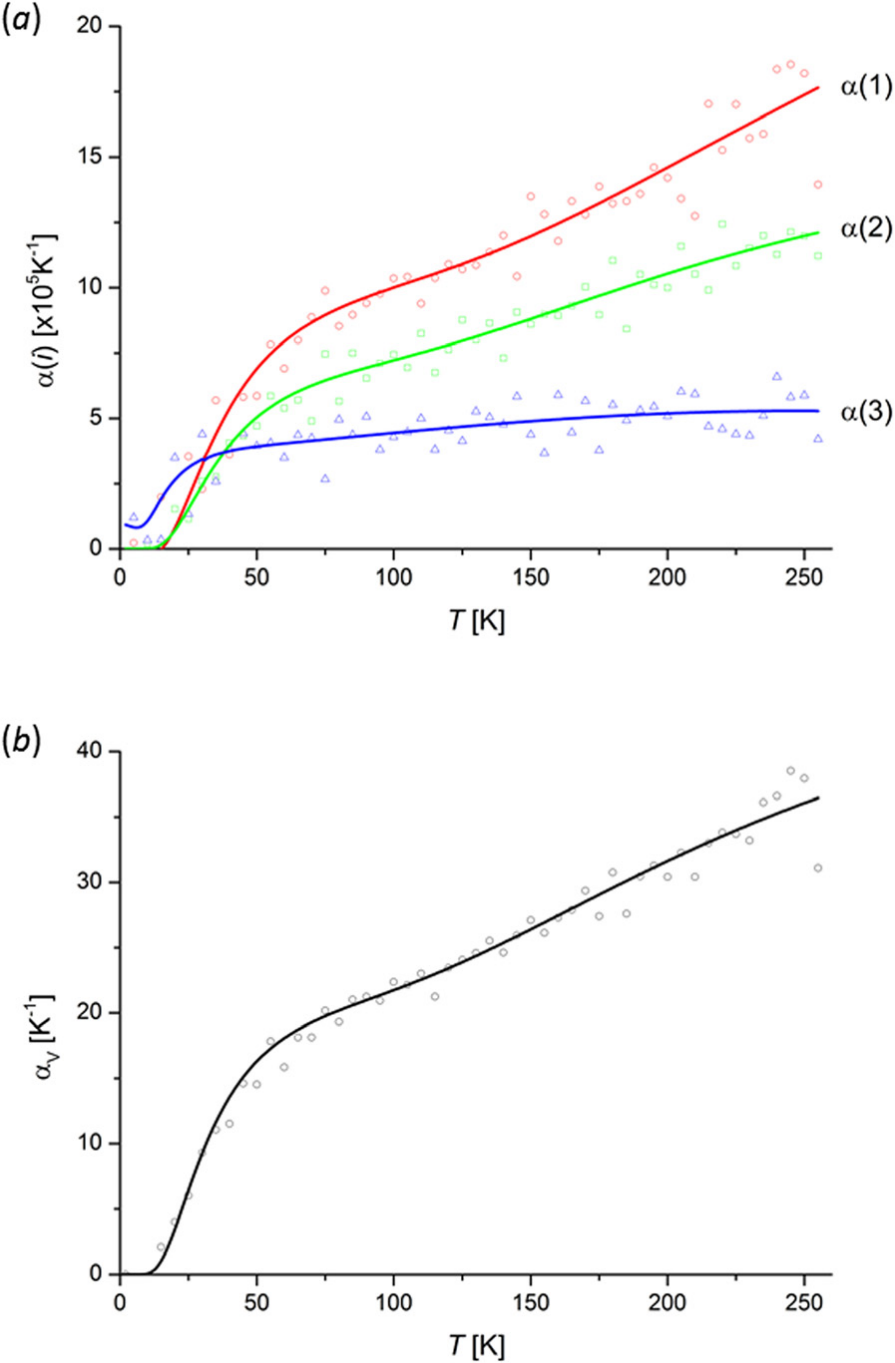
Thermal expansion of piperidine. (***a***) Principal values of the thermal expansion tensor: (***b***) The volume thermal expansion. In each case the small circles are calculated from pairs of experimental data points, whereas the trend-line is calculated from pairs of points with values taken from the Einstein fits in Figure 5. At very low temperature, the points are calculated from very small differences, and the results less reliable than at higher temperature.

The thermal expansion tensor was obtained by dividing the strain tensor evaluated for pairs of neighbouring temperature points by the temperature increment. This procedure yields identical diagonal strain tensor elements as given by Schlenker *et al.* [60] The off-diagonal term is approximately equal to Schlenker *et al.*’s formula for small changes in β. The tensors so-obtained were diagonalised using the JACOBI routine from *Numerical Recipes* [48]. The data are shown in Figure 7a, with the trend-line obtained in the same way using ideal values of the cell dimensions from the double-Einstein fitting described above. The largest component [α(1)] is approximately aligned with a*, the mean angle being 8° between 32 and 225 K. α(3), the smallest component is approximately aligned along c*. One axis, in this case α(2), must lie along **b** by symmetry. Though α(3) tends towards a constant value at 255 K, all three curves have positive slopes at the melting point, suggesting that new vibrational energy levels are being accessed throughout the temperature range studied. At 255 K the expansion coefficients measured from the trend-lines are 17.3(3), 12.1(2) and 5.5(3) × 10^−5^ K^−1^ for α(1)-α(3), respectively. The increase in unit cell volume between 2 K and 255 K is 31.16 Å^3^ which represents 5.9% of the volume at 2 K; the variation of the volume expansion coefficient is shown in Figure 7b.

Structures of piperidine were optimised by periodic DFT using cell dimensions fixed to the experimentally-determined values at 2 K and in 25 K steps from 25 K to 250 K. The resulting structures were then combined into a movie, available in the Additional file 2: Movie S1, enabling the thermal expansion to be visualised. The H-bonded chains are arranged in rows which run parallel to **c**; these rows move apart as temperature is increased. Interestingly, the largest expansion component is the one approximately aligned with the axis (*a*) associated with the largest Einstein temperatures given in Table 3; similarly the smallest component lies approximately along *c*, which is associated with the smallest Einstein temperatures. In part, this may be the result of applying Equ. 5 to cell dimension data, when it strictly applies only to volume. However, the strain tensors components obtained from the variable temperature and pressure experiments adopt a similar alignment, suggesting that the anisotropy of thermal expansion is determined by the compressibility of the structure along different directions (see below).

The constant-pressure heat capacity (*C*_p_) of crystalline piperidine-h_11_, which has been measured as a function of temperature [52], can be converted to the constant-volume heat capacity (*C*_v_) using Equ. 7 [58,59]:

7 $$ {\mathit{\mathsf{C}}}_{\mathsf{v}}={\mathit{\mathsf{C}}}_{\mathsf{p}}-{\alpha}^{\mathsf{2}}\mathit{\mathsf{T}}\mathit{\mathsf{V}}\mathit{\mathsf{B}} $$

**Figure 8:**
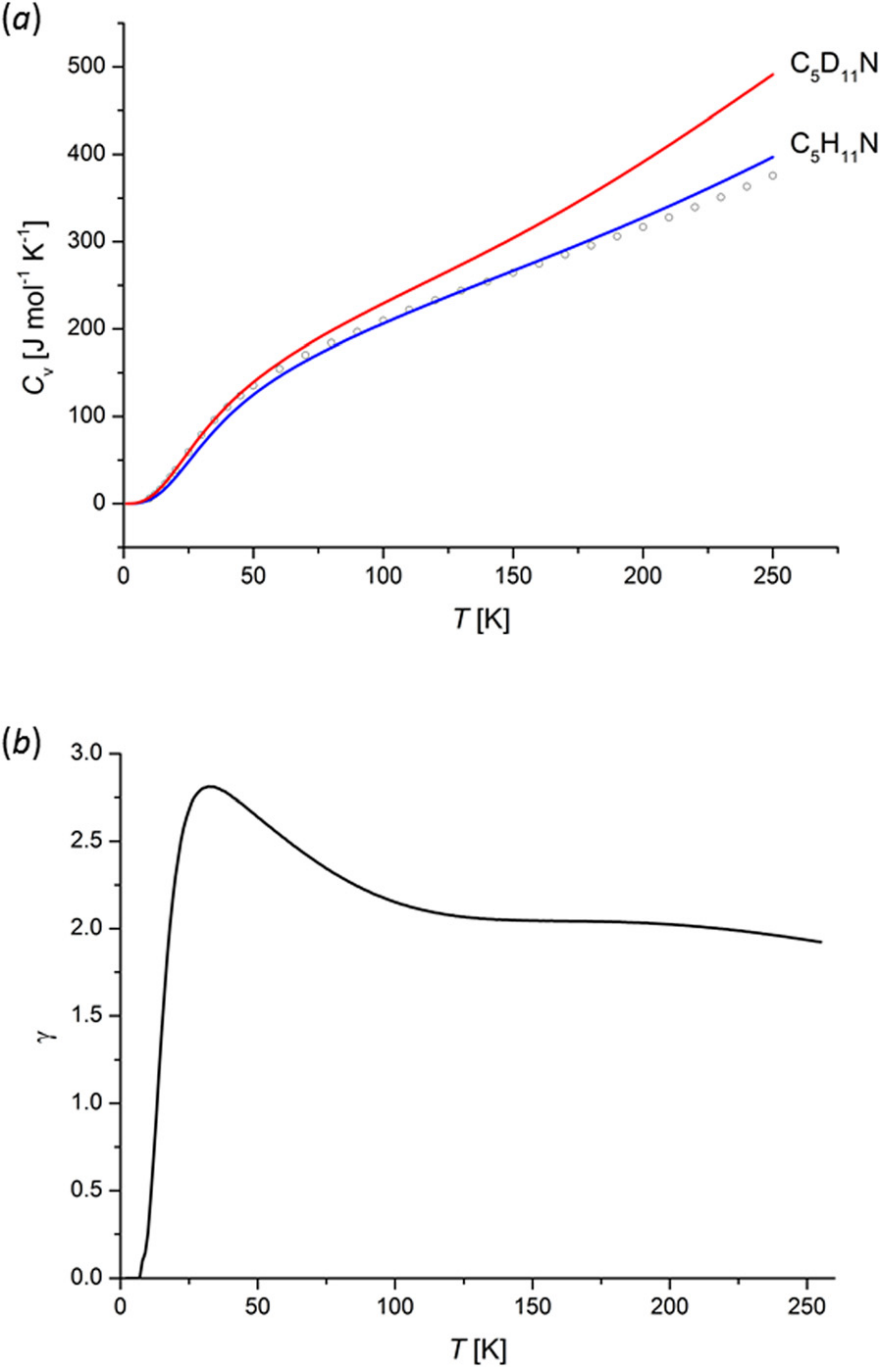
Thermodynamic parameters of crystalline piperidine. (***a***) The constant-volume heat capacities of piperidine-h_11_ (blue) and piperidine-d_11_ (red). The circles are calculated from experimentally-determined *C*_p_ values measured for the h_11_ isotopologue, and the solid blue line is determined from a DFT phonon calculation. The solid red line was obtained from a similar calculation on the d_11_ system. (***b***) Variation of the Grüneisen parameter with temperature.

The values of *C*_v_ determined in this way agree well with those derived from the phonon density of states calculated by periodic DFT on C_5_H_11_N (Figure 8a). Values of *C*_v_ were therefore calculated in the same way for C_5_D_11_N. The results are also shown in Figure 8a, the generally increased values being the result of the smaller vibrational frequencies for the deuterated isotopologue. The calculated heat capacities were combined with data for the variation of *V* and the thermal expansion coefficient with temperature, and the bulk modulus determined from the high-pressure measurements described below, to obtain the temperature dependence of the Grüneisen parameter, γ, defined in Equ. 1. The results (Figure 8b) show that γ, which measures the variation of vibrational frequencies with temperature, initially increases to reach a maximum of 2.8 at about 30 K, but thereafter drops back to a value near 2. The drop-off arises because external vibrational modes are populated below 100 K, and these have a greater sensitivity to temperature than the internal vibrational modes which are excited above 100 K [61]. Note that in this analysis the bulk modulus is assumed to be constant with temperature.

### The effect of pressure on the unit cell dimensions of piperidine

**Figure 9:**
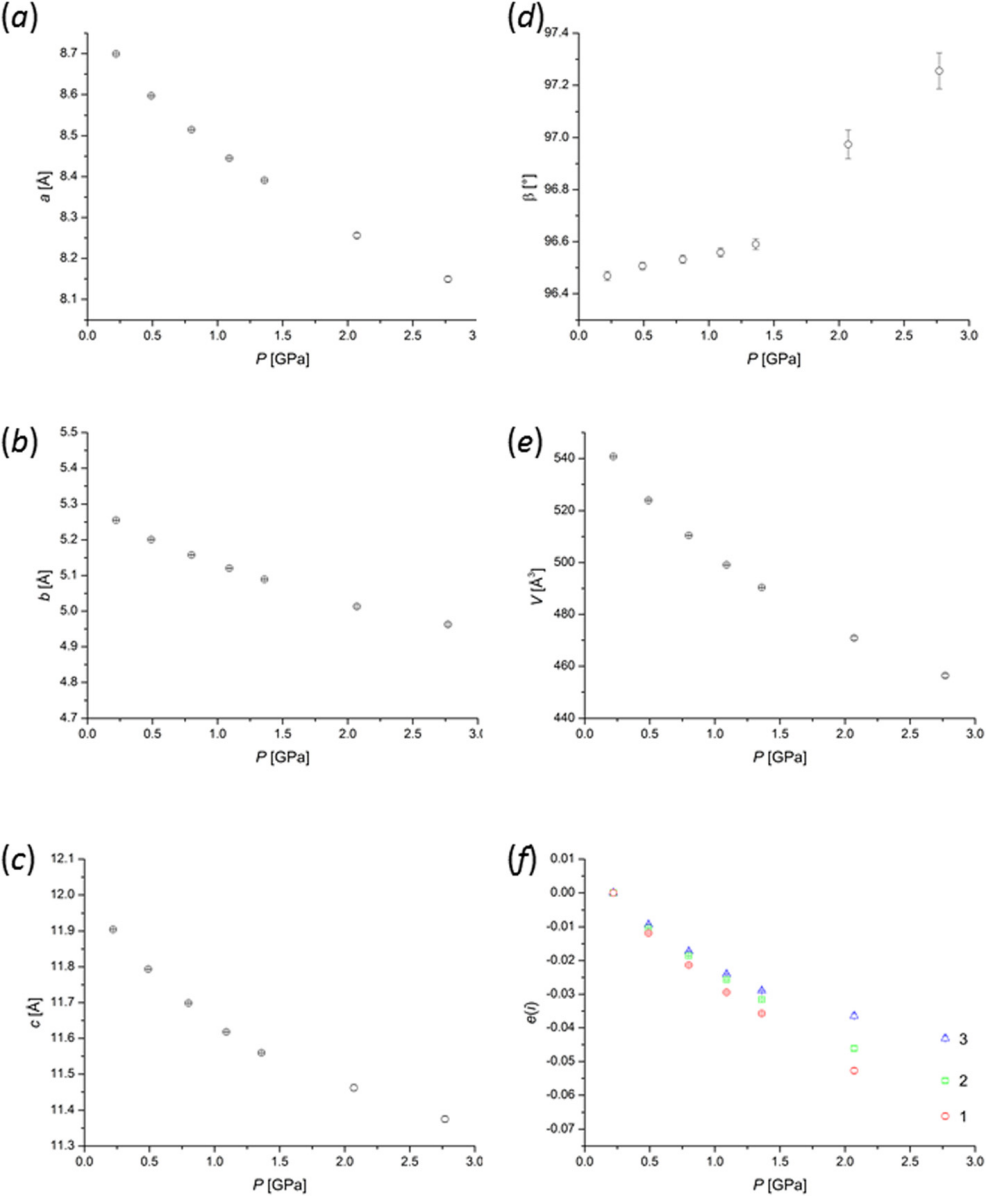
Variation of the lattice parameters with pressure. (***a***) – (***d***) show the trends of the *a*, *b* and *c*-axis lengths and the β-angle with pressure. (***e***) shows the change in volume and (***f***) the principal axes of the strain tensor calculated using the cell dimensions at 0.22 GPa as a reference.

Piperidine-d_11_ crystallises at room temperature at a pressure of 0.22 GPa. The crystal structure obtained is the same as that described above, with a slightly greater volume than at 2 K. The effect of increasing pressure on the cell parameters and volume is shown in Figure 9a-e, the volume reducing by 15.5% up to 2.77 GPa. The *a* axis undergoes the greatest reduction in length (6.2%) between 0.22 GPa and 2.77 GPa. The *c* axis is the least sensitive to pressure, reducing by 4.5%. The principal axes of the strain tensor calculated between 0.22 and 2.77 GPa, shown in Figure 9f., are similar in direction to those determined at ambient pressure between 2.5 and 255 K (Table 4). The strain is also more isotropic under compression, particularly so at low pressures (Figure 9f and Table 4).

**Table 4:** **Comparison of eigenvalues and eigenvectors of the temperature and pressure induced strain tensors**

***T*** **(255 K** ***versus*** **2 K)**		***P*** **(0.22 GPa** ***versus*** **0.80 GPa)**	
**Eigenvalue**	**Eigenvector**	**Eigenvalue**	**Eigenvector**
−0.026281(14)	[0.113 0.000 0.021]	−0.0213(3)	[0.115 0.000 0.016]
−0.019036(10)	[0.000 0.188 0.000]	−0.0186(2)	[0.000 0.190 0.000]
−0.011088(16)	[−0.016 0.000 0.081]	−0.01725(17)	[−0.009 0.000 0.083]

The volume decrease between 255 and 2 K (31.2 Å^3^) is similar to that between 0.22 and 0.80 GPa (30.3 Å^3^). Eigenvector components are given with respect to the direct crystallographic axes at 2 K and 0.22 GPa.

The bulk modulus (*B*) refined for a third order Birch-Murnaghan equation of state [14] is 6.4(4) GPa, though the data-set used to calculate this quantity is admittedly rather limited. The values of *V*_0_ and *B*’ refined to 557.0(15) Å^3^ and 8.2(8) respectively (χ^2^ = 0.74). Molecular solids typically have *B* < 30 GPa and the following *B* values are useful for comparison [62]: Ru_3_(CO)_12_ 6.6 GPa, *L*-alanine [63] and salicylaldoxime [64] 13.3 GPa, NaCl 25 GPa, quartz 37 GPa, ceramics 50–300 GPa and diamond 440 GPa. Piperidine is thus quite soft compared to other hydrogen bonded materials.

### The effect of pressure on the structure of piperidine

The neutron powder diffraction data obtained at elevated pressure were of medium resolution and modest statistical precision, and during structure refinement the piperidine molecules were treated as rigid bodies with bond distances and angles taken from the X-ray structure at 150 K. To test the validity of the rigid-body assumption, the experimental structures were optimised using periodic DFT, holding the unit cell dimensions fixed to experimental values. Such calculations have been shown to reproduce experimentally determined atomic positions with crystal packing similarity values^a^ of < 0.1 Å [65]. For piperidine, crystal packing similarities [43,66] of the experimental and optimised structures were between 0.021 and 0.037 Å, confirming that the rigid-body assumption is reasonable.

**Figure 10:**
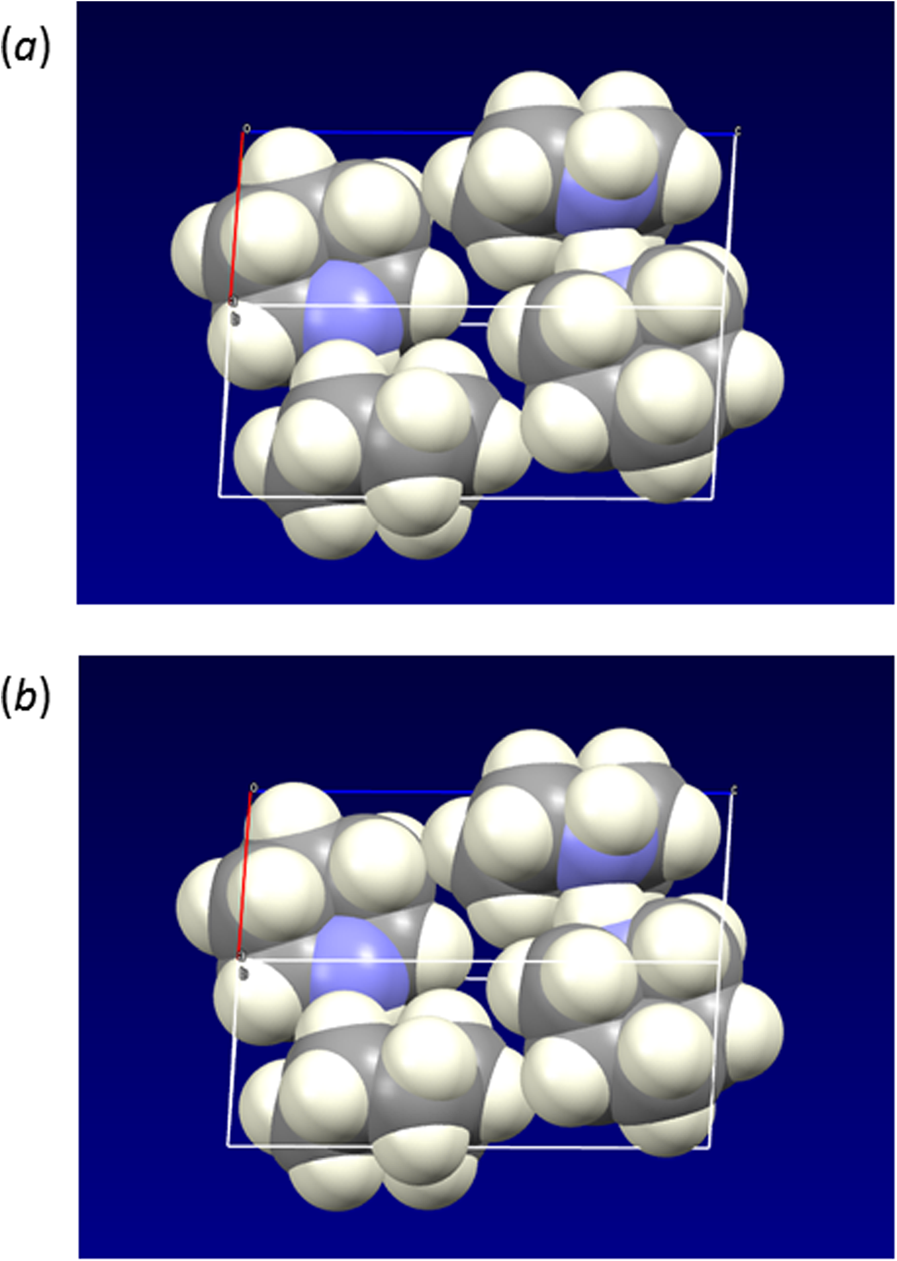
Interstitial voids at (***a***) 0.22 and (***b***) 1.09 GPa. Images were generated using the experimentally determined coordinates; a movie extended to 2.77 GPa using the DFT optimised coordinates is available in the Additional file 3: Movie S2b).

A movie showing the effect of pressure, generated using optimised structures in the same way as the movie for temperature described above in “The effect of temperature on the structure of piperidine”, is available in the Additional file 3: Movie S2a). Comparison of the pressure and temperature series movies shows the more nearly isotropic distribution of strain as well as more prominent changes in the orientations of the molecules under compression. The largest interatomic distance change occurs for C5H10…H3, which measures 3.04 Å at 0.22 GPa and 2.51 Å at 1.09 GPa, a reduction of 17.4%. Other contacts listed in Table 2 change by between 4 and 8%; the hydrogen bonds shorten from 2.18 Å at 0.22 GPa to 2.09 Å at 1.09 GPa. The overall effect is to fill the rather large interstitial voids formed between the H-bonded chains (Figure 10, Additional file 3: Movie S2b).

Over the course of the first one or two kbars of applied pressure, provided the symmetry remains unchanged and volume changes are small, the path of compression might be expected to follow the path of a low-energy lattice vibration. Specifically, the mode would be expected to lie at the Γ-point and belong to the totally symmetric representation, in the present case, where the lattice point symmetry is 2/*m*, *A*_1g_. The lowest totally symmetric phonon, which has a calculated frequency of 36 cm^−1^ is shown as an animation in the Additional file 4: Movie S3a) and can be compared to a sequential animation of the structures obtained at 0.22 and 0.49 GPa (Additional file 4: Movie S3b). The similarity between the two animations is striking, suggesting that structure determinations at pressures of a few kbar can potentially be used to visualise low-frequency phonons.

### Comparison of the effects of pressure and temperature

The strain induced by cooling or compression is characterised by similarly orientated strain tensors, with largest principal axis lying approximately along **a**, the middle lying exactly along **b** and the smallest approximately along **c** (Figure 3c). Though the principal axes are similarly orientated, the tensor describing the response to pressure is more isotropic.

In an attempt to gain some insight into the structural reasons for these similarities and differences the DFT-optimised structure at 0.22 GPa was reoptimised with first the *a* and then the *c* axis reduced in length by 5%. PIXEL calculations were then carried out on all three optimised structures, referred to as ‘opt’, ‘A’, and ‘C’ respectively. The lattice energies were −55.2, −53.8 and −53.1 kJ mol^−1^, respectively; though the differences between these figures are small, they are consistent with the greater compressibility along **a**. The H-bond energies (kJ mol^−1^) are −21.7 (opt), −21.4 (A) and −21.9 (C), indicating that compression along **c** actually stabilises the H-bonds, while compression along **a** destabilises them. The H-bond distances (Å) are opt 2.136, A 2.074 and C 2.111, and though in both A and C the electrostatic term is more negative as a result of the shorter N…H distance, it is outweighed by a more positive repulsion term in A.

**Figure 11:**
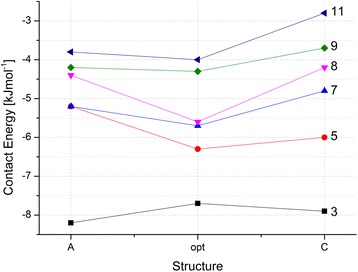
The effects of a 5% reduction in the ***a*** and ***c*** axis lengths on intermolecular contact energies. The energies were calculated for different model structures using the PIXEL method. Model ‘opt’ refers to the structure at 0.22 GPa after optimisation of the coordinates using DFT (holding the cell dimensions fixed). Model ‘A’ was obtained from model ‘opt’ by reducing the length of the *a*-axis by 5% and re-optimising the structure. Model ‘C’ was obtained in a similar way, but after reducing the length of the *c*-axis by 5%. The Figures 3 and 5 *etc.* on the right of the figure refer to the contact numbers listed on the far left of Table 2. Contacts 4, 6, 10 and 12 are symmetry equivalent to those shown. The H-bond contacts 1 and 2 are omitted for clarity.

The energies of other contacts are depicted in Figure 11. In all but one case (#5), the contacts in structure C are less energetic that those in A, indicating that compression along **c** destabilises the inter-chain van der Waals interactions more than compression along **a**. While this explains the greater compressibility along the *a*-direction, it is also clear that the balance is a rather fine one, and this is manifested in the variable pressure series by the more isotropic strain tensor. As pressure increases, the *PV* contribution to free energy becomes appreciable. As Figure 10 shows, closure of the interstitial voids involves a significant component of *c*-axis shortening, and the isotropy of strain at high pressure is the result of compression along **a** being favoured by the inter-chain contacts, while compression along **c** leads to greater volume reduction.

## Conclusions

We have explored the crystal structure of piperidine under a range of different conditions of temperature and pressure. Although a previous study had indicated another phase exists, no new phases have been identified here, and it is possible that the alternative phase identified by Parkin on the basis of cell dimensions was a metastable form. The crystal structure of piperidine is characterised by chains of molecules linked by NH…N hydrogen bonds which are disposed about a 2_1_ axis running along **b**. The unit cell of the metastable phase has a *b* axis length very similar to that of the *b*-axis of the phase discussed in this paper, and both phases are likely to feature similar H-bond chains. Though our original crystal growth experiments and DSC measurements indicated formation of a metastable form, there is no mention of transitions in the report of heat capacity measurements in ref. [52], and it is possible that its formation is dependent on rather specific crystallisation conditions which remain unidentified. Deuteration is also known to influence formation of alternative phases [67].

Though the H-bonds have a characteristic and easily recognisable structural signature, the same is not true of van der Waals interactions [68], but their influence can be appreciated with the aid of packing energy calculations, such as the PIXEL method used here. In the case of piperidine, though H-bonds are the most energetic contacts, around 60% of the sublimation enthalpy is the result of van der Waals interactions in which individual interatomic distances teeter on the brink of insignificance when assessed in the conventional manner using van der Waals radii.

Periodic DFT calculations reproduce the experimental cell dimensions of piperidine at 2 K to within 1%. The phonon frequencies calculated using this optimised structure, though they are obtained on the basis of a harmonic model for the energies of small atomic displacements, also reproduce experimental heat capacity data well. These calculations enable the thermal expansion of piperidine to be understood in terms of a simple quantitative model for how energy in the form of heat is absorbed into lattice vibrations.

The response of piperidine to pressure is superficially similar to its response to cooling. Although the principal directions of strain are essentially the same under both sets of varying conditions, the tensor describing the strain developed under pressure is more isotropic. PIXEL calculations demonstrate that the energetic imbalance between the weak van der Waals interactions and the relatively strong H-bonds determines the anisotropy of the strain tensor on cooling. Under pressure the need to fill space efficiently competes with minimisation of free energy though intermolecular contacts, making the strain less anisotropic.

### Endnote

^a^This is a parameter quantifying the similarity of two crystal structures, and it is defined as the root-mean square deviation calculated for a cluster of 15 molecules taken from each structure. Crystal packing similarities were calculated in MERCURY.

## Additional files

Additional file 1: Table S1. Unit cell parameters between 2 and 255 K. Additional file 2: Movie S1. Visualisation of thermal expansion in steps of 25 K. Coordinates were generated by geometry optimisation with periodic DFT holding the cell dimensions fixed at experimentally-determined values. Additional file 3: Movie S2. Visualisation of the compression series between 0.22 and 2.77 GPa. Coordinates generated as in Movie S1 in (a) ‘stick’ format and (b) space-filling format to show interstitial void space. Additional file 4: Movie S3. (a) Experimental structures at 0.22 and 0.49 GPa. (b) Animation of the lowest frequency totally symmetric Γ-point phonon.
