# Supplementary figures and images for: The effect of temperature and pressure on the crystal structure of piperidine
Source: Chem Cent J. 2015 Apr 12;9:18. doi: 10.1186/s13065-015-0086-3 (PMC4403828; doi:10.1186/s13065-015-0086-3)

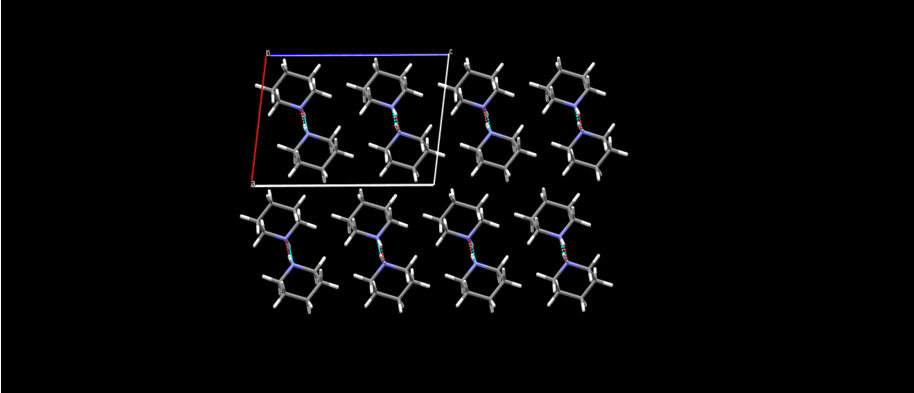

Supplement: Additional file 3: Movie S2. — Visualisation of the compression series between 0.22 and 2.77 GPa. Coordinates generated as in Movie S1 in (a) ‘stick’ format and (b) space-filling format to show interstitial void space. [file 13065_2015_86_MOESM3_ESM.zip › Additional file 3/5856532181478788_add4.gif]

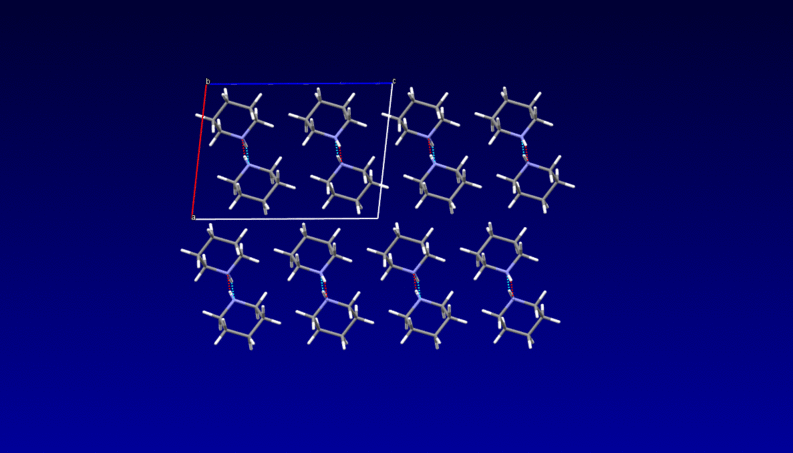

Supplement: Additional file 3: Movie S2. — Visualisation of the compression series between 0.22 and 2.77 GPa. Coordinates generated as in Movie S1 in (a) ‘stick’ format and (b) space-filling format to show interstitial void space. [file 13065_2015_86_MOESM3_ESM.zip › Additional file 3/5856532181478788_add5.gif]

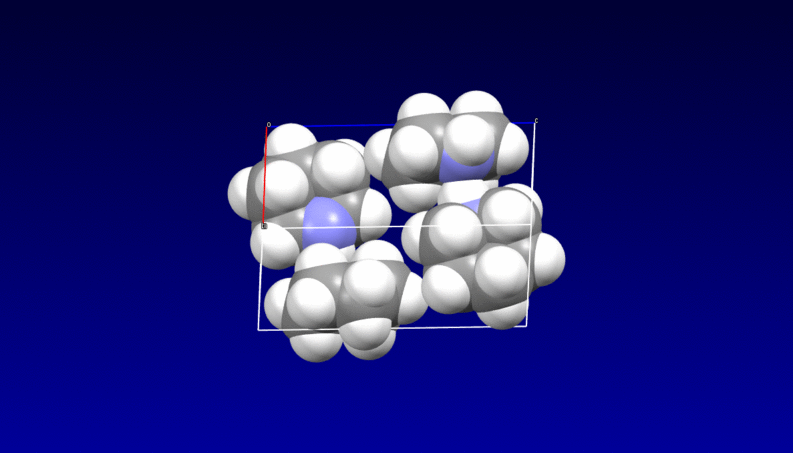

Supplement: Additional file 3: Movie S2. — Visualisation of the compression series between 0.22 and 2.77 GPa. Coordinates generated as in Movie S1 in (a) ‘stick’ format and (b) space-filling format to show interstitial void space. [file 13065_2015_86_MOESM3_ESM.zip › Additional file 3/5856532181478788_add6.gif]

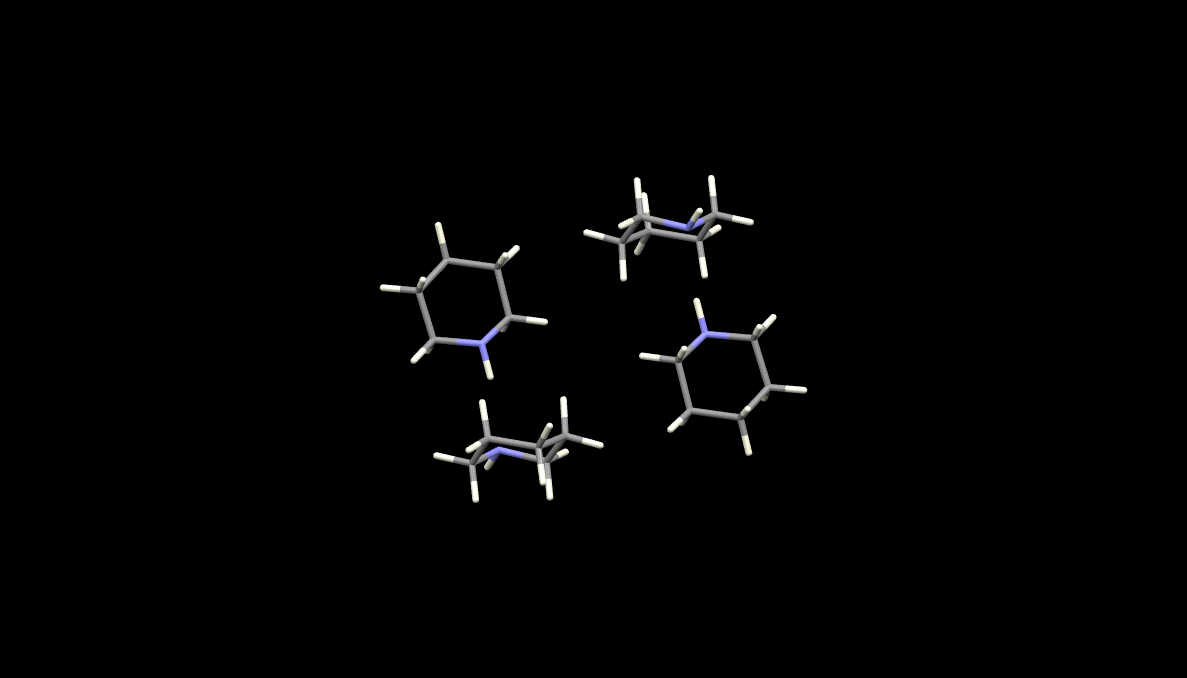

Supplement: Additional file 4: Movie S3. — (a) Experimental structures at 0.22 and 0.49 GPa. (b) Animation of the lowest frequency totally symmetric Γ-point phonon. [file 13065_2015_86_MOESM4_ESM.zip › Additional file 4/5856532181478788_add7.gif]

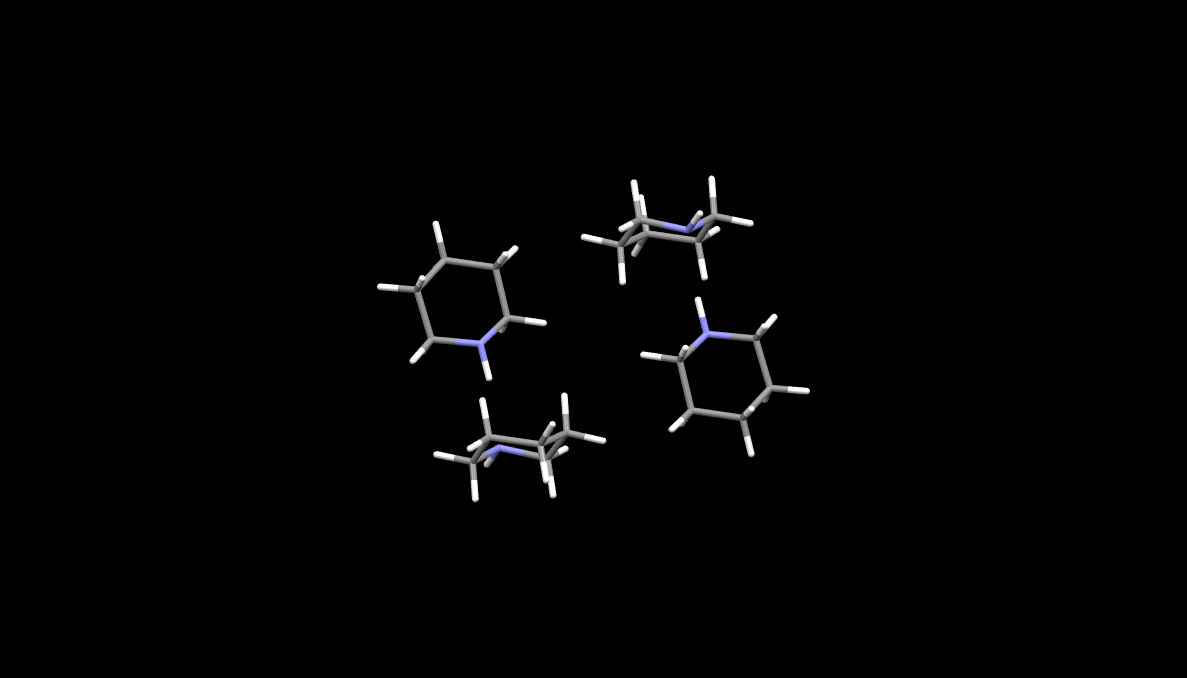

Supplement: Additional file 4: Movie S3. — (a) Experimental structures at 0.22 and 0.49 GPa. (b) Animation of the lowest frequency totally symmetric Γ-point phonon. [file 13065_2015_86_MOESM4_ESM.zip › Additional file 4/5856532181478788_add8.gif]
